# Striking Back against Fungal Infections: The Utilization of Nanosystems for Antifungal Strategies

**DOI:** 10.3390/ijms221810104

**Published:** 2021-09-18

**Authors:** Wei Du, Yiru Gao, Li Liu, Sixiang Sai, Chen Ding

**Affiliations:** 1 College of Life and Health Sciences, Northeastern University, Shenyang 110015, China; 1710064@stu.neu.edu.cn (W.D.); 1910449@stu.neu.edu.cn (Y.G.); 1901376@stu.neu.edu.cn (L.L.); 2 School of Medicine, Binzhou Medical University, Yantai 264003, China

**Keywords:** fungal infection, antifungal therapies, nanosystems

## Abstract

Fungal infections have become a major health concern, given that invasive infections by *Candida, Cryptococcus,* and *Aspergillus* species have led to millions of mortalities. Conventional antifungal drugs including polyenes, echinocandins, azoles, allylamins, and antimetabolites have been used for decades, but their limitations include off-target toxicity, drug-resistance, poor water solubility, low bioavailability, and weak tissue penetration, which cannot be ignored. These drawbacks have led to the emergence of novel antifungal therapies. In this review, we discuss the nanosystems that are currently utilized for drug delivery and the application of antifungal therapies.

## 1. Fungal Infection

Fungal infections are a major threat to humans and number in the billions, leading to more than 1.5 million deaths annually [1,2,3]. *Candida*, *Aspergillus,* and *Cryptococcus*, are the major pathogenic fungi in humans, causing 90% of the deaths in both immunocompetent and immunocompromised individuals [2,3]. Other pathogenic fungi, including *Pneumocystis, Coccidioides,* and *Histoplasma,* can also cause severe tissue damage and even death (Table 1).

The type of fungal infection is highly dependent on the fungal species and the immune status of the hosts [4]. For instance, superficial infections in humans are among the most common fungal infections, occurring in over 1 billion people. Over 135 million women are diagnosed with *Candida* mucosal infections [5]. However, the most devastating fungal infections are invasive. These are initiated by the inhalation or inoculation of fungal spores or by an imbalance of the commensal fungi of the host. Invasive candidiasis, aspergillosis, and cryptococcosis occur in the bloodstream and deep-seated organs as a result of fungi from the genera *Candida, Aspergillus,* and *Cryptococcus,* respectively. Additionally, fungal infection also results in or enhances severe host allergic responses, including asthma, cystic fibrosis, or chronic nasal sinus symptoms [6,7,8,9,10].

*Candida* spp. make up the commensal fungi residing within human superficial, mucosal, or intestinal tract regions, and their colonization and growth are highly restricted when the host is immunocompetent. Studies demonstrate that over 15 distinct *Candida* species are pathogenic fungi, of which five species cause the most invasive infections in humans: *Candida albicans, Candida krusei, Candida glabrat, Candida tropicalis,* and *Candida parapsilosis* [11,12,13,14]. Recent studies show that *Candida auris* has emerged globally as a multidrug-resistant fungal pathogen that leads to significant patient mortality [15,16,17,18]. In addition to *Candida*, *Cryptococcus,* and *Aspergillus* lead to severe lung infections and can lead to fatal infections, including pneumonia and meningocephalitis.

As with candidiasis, cryptococcosis is also a globally distributed invasive fungal infection caused by *Cryptococcus* species and leads to significant mortality and therapeutic challenges. *Cryptococcus* was first identified in 1894 from the tibia of a 31-year-old woman, and cryptococcosis has been attributed to a single fungal species *Cryptococcus neoformans*. The cryptococcosis epidemic is highly consistent with the AIDS pandemic of the 1980s [23,24,25,26,27]. However, because molecular technology and epidemic research have improved, *C. neoformans* var. *gattii* was classified as a distinct species, *C. gattii,* in 2002. This species has been considered the causative fungi for the outbreak of cryptococcosis in the North American Pacific Northwest in 1999 [28,29,30,31,32,33,34,35,36]. 

Ecologically, cryptococci reside in various tree species, especially the waxier cuticles, while *C. neoformans* is particularly abundant in pigeon excreta [25,37]. These two cryptococci can also survive and replicate in soil, amoebae, and vertebrates [38]. Furthermore, they have developed sophisticated strategies, such as thermo-tolerance, pH-tolerance, and resistance to phagocytosis from host immune cells, which facilitate fungal growth and persistence within environmental niches and vertebrates [39,40,41,42,43]. These strategies endow cryptococci with growth advantages, including severe virulence. Cryptococcal infection begins with the inhalation of cryptococci spores into the lungs and can cause pneumonia in immunosuppressed patients. However, these fungal cells establish an asymptomatic latent infection in immunocompetent hosts, where the colonizing fungal cells can disseminate to other tissues, especially the central nervous system, which occurs through uncharacterized mechanisms [44,45]. Once the brain has been colonized, cryptococcosis leads to a devastating infection of the meninges and lethal meningoencephalitis [46].

*Aspergillus* is a saprophytic fungus found in soil and comprises at least 200 species. *Aspergillus* spp. is common and isolated from cultures of the respiratory tracts of asymptomatic patients who lack evidence of invasive or allergic disease [47,48,49]. Moreover, aspergillosis is highly associated with chronic obstructive pulmonary disease [50]. *Aspergillus* spp. is much more common than other fungi, and approximately 37% of healthy adults carry these fungi [51]. However, they are not always associated with infection. Nevertheless, colonization by *Aspergillus* spp. is associated with increased risk of invasive infections across a wide range of immunocompromised individuals [52]. To date, *Aspergillus* pathogens include *A. fumigatus, A. flavus, A. niger, A. terrus, A. clavatus,* and *A. nidulans,* the first of which is the most common. 

Distinct from *Cryptococcus*, however, *Aspergillus* can produce small asexual spores, called conidia, which are 2–3 μm in diameter. The dispersal of *A. fumigatus* produces conidia that are hydrophobic and can spread and sustain for a much longer time in the air, making it a highly virulent fungus [53,54,55]. In healthy individuals, epithelial cells in the airway and alveolar macrophages efficiently eliminate inhaled conidia, but resident conidia can evade mucocilliary clearance and lodge in the alveoli deep in the lungs, germinating into short hyphae in less than 6 h [56]. Aside from host immune status, *Aspergillus* colonization is related to certain genetic factors of the host. Impaired expression of the transcriptional factor *ZNF77* in bronchial epithelia can result in the destruction of epithelial cell integrity, and upregulation of extracellular matrix proteins has been shown to facilitate conidial adhesion [57].

## 2. Conventional Treatments for Candidiasis, Cryptococcosis, and Aspergillosis

Researchers have developed several antifungal treatments to contend with the increasing challenge of fungal infections. However, the significant side effects and development of drug resistance in fungi have not been adequately addressed [58,59,60,61].

All fungal cells produce cell walls that are based on aminopolysaccharide structures and referred to as the chitin layer. This layer has slight differences between yeasts and filamentous cells, which contain matrices of β-1, 3-glucans plus either β-1, 6-glucans or α-1, 3-glucans, respectively [62,63,64,65]. 

**Figure 1:**
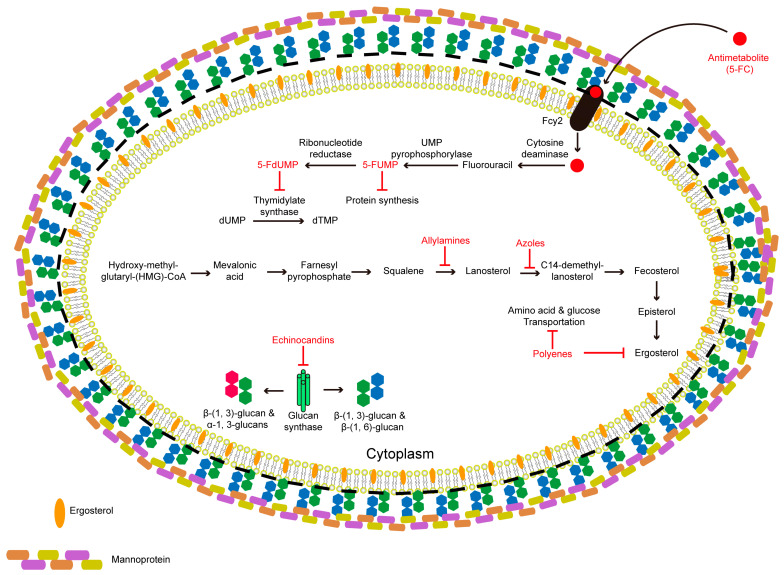
Schematic representation of the conventional antifungal agents, their targets, and actions. Antimetabolite, 5-Fluorocytosine (5-FC), is a fluorinated pyrimidine analog with fungicidal activity via interfering the pyrimidine metabolism, RNA/DNA and protein synthesis. First, 5-FC is taken up by fungal cells via a cytosine permease (encoded by gene *FCY2*) and is converted to 5-fluorouracil (5-FU), and is then transformed by UMP pyrophosphorylase into 5-fluorourdine monophosphate (5-FUMP). Then, 5-FUMP is incorporated into RNAs to inhibit the protein synthesis. Additionally, ribonucleotide reductase enables the conversion of 5-FUMP into 5-fluorodeoxyuridine monophosphate (5-FdUMP), a potent inhibitor of thymidylate synthase that inhibits fungal DNA synthesis and nuclear division. Azoles are inhibitors for cytochrome P450-dependent enzyme lanosterol 14α-demethylase (CYP51) encoded by the *ERG11* gene, and thus block the conversion of lanosterol to ergosterol. Allylamines block ergosterol biosynthesis via inhibiting squalene epoxidase (*ERG1*) that lead to squalene accumulation and increased permeability may cause the disruption of cellular organization. Echinocandins act as noncompetitive inhibitors of β-(1, 3)-d-glucan synthase enzyme complex and leads to disruption of the cell wall structure, resulting in osmotic instability and fungal cell death. Polyenes specifically bind to the lipid bilayer and form a complex with the ergosterol producing pores that leads to the disruption of the cell membrane, leakage of the cytoplasmic, contents and oxidative damage in fungal cells. Amphotericin B (AmB) binds ergosterol and forms an extra-membranous fungicidal sterol sponge destabilizing membrane function.

In addition, yeast cells construct galactosaminoglycans and protein-based outer layers, and galactomannan is an additional element in the final layer of filamentous cells. Moreover, fungal cells utilize ergosterol rather than cholesterol to manufacture cell membranes [66,67]. These elements of fungal cell walls and membranes distinguish invasive fungi from host mammalian cells and act as therapeutic targets for antifungal drugs (Figure 1).

Common clinical antifungal drugs have distinct molecular targets and can be divided into 5 categories (Figure 1, Table 2): (i) azoles, (ii) polyenes, (iii) echinocandins, (iv) allylamines, and (v) antimetabolites. Azoles are chemically synthesized small molecules that primarily block the ergosterol synthesis pathway by inhibiting lanosterol 14α-demethylase (Erg11), which is essential for fungal cell membrane formation. They have been used as antifungal drugs since the 1970s, when they were found to impart a broad spectrum of activity against various fungal species.

Polyenes were isolated from *Streptomyces* spp., where they have functions in the bacterial defense mechanism. This class of drug mainly sequesters ergosterol and disrupts the fungal cell membrane via pore formation, resulting in leakage of cytoplasmic contents and fungal cell death [95,96]. The most potent, amphotericin B (AmB), is the most common polyene used for invasive fungal infections by forming an extra-membranous fungicidal sterol sponge that destabilizes membrane function [97]. In contrast with other kinds of polyenes, natamycin (NAT) inhibits fungal growth by reversibly inhibiting the amino acid and membrane transport proteins without altering the cell membrane permeability [85]. 

Enchinocandins target β-1, 3-glucan synthase and negatively impact fungal cell wall integrity. These antifungal agents have good safety profiles, but have poor oral bioavailability, due to the lipid side chains. They have efficient therapeutic applications against both the planktonic cells of *Candida* and their biofilm formation. Additionally, this antifungal agent has been used to treat aspergillosis [98,99]. 

Allylamines inhibit squalene epoxidase activity and destroy the ergosterol synthesis pathway [100]. The fifth antifungal category agent is the antimetabolite 5-fluorocytosine (5-FC), which acts as a nontoxic prodrug and enters into fungal cells via the cytosine permease Fcy2. Furthermore, 5-FC can be converted into toxic 5-fluorouracil (5-FU) by cytosine deaminase Fcy1, which is only present in fungal cells. The UMP pyrophosphorylase transforms 5-FU to 5-fluorourdine monophosphate (5-FUMP), which incorporates into RNA and replaces UTP, thus inhibiting protein synthesis. Next, ribonucleotide reductase catalyzes 5-FUMP to 5-fluoro-2′-deoxyuridine-5′-monophosphate (5-FdUMP), which acts as a thymidylate synthase inhibitor and results in inhibition of fungal RNA and DNA synthesis. 

## 3. Unsatisfactory Properties of Currently Used Antifungal Drugs

The five classes of conventional antifungal drugs have been determined to have great efficiency for treating both superficial and invasive fungal infection. Nevertheless, their side effects and unpleasant properties highly restrict their applications. As the most commonly used antifungal drugs in clinical practice, the major concerns of using azoles are their interactions with drugs that act as substrates for cytochrome P450, leading to off-target toxicity and fungal resistance to azoles [101,102]. Polyenes target fungal ergosterol, which is structurally similar to mammalian cholesterol. As a result, AmB displays devastating nephrotoxicity and infusion-related reactions [103,104]. As a result, its dosage is highly restricted, and it is usually replaced by an azole drug (voriconazole). Rather than invasive fungal infections, allylamines are normally used for treating superficial fungal infection, such as onychomycosis, which occurs in the fingernails or toenails [105].

As a highly effective antifungal agent, antimetabolite 5-FC is severely hepatoxic and results in bone-marrow depression [106,107,108]. Additionally, monotherapy with 5-FC triggers significant fungal resistance. Its primary clinical use is in combination with AmB for severe cases of candidiasis and cryptococcosis [109,110]. Although several effective antifungal agents have been prescribed for decades, their therapeutic outcomes remain unsatisfactory. Aside from these traditional antifungal agents being highly toxic, fungi tend to become resistant to them. Moreover, these antifungal agents display distinct efficiencies in tissue penetration and oral bioavailability. 

In general, fluconazole, 5-FC, and voriconazole are small molecules and display better tissue penetration than the larger, more lipophilic agents (itraconazole) and amphipathic agents (AmB and echinocandins). Additionally, AmB and echinocandins exhibit delayed drug metabolism and accumulate in tissues [111]. Current strategies for improvement include developing analogs of these compounds, evaluating current drugs for their potential antifungal effects, finding new targets for antifungal drugs, and determining new fungal antigens as vaccine candidates [112,113].

Another possible strategy is using nanotechnology to modify or encapsulate currently used antifungal agents to improve their efficacy. To date, several nanomaterials have been investigated and presented as innovative antifungal agents, which include biodegradable polymeric and co-polymeric-based structures, metallic nanoparticles, metallic nanocomposites, and lipid-based nanosystems [114,115,116]. Additionally, the size range of nanoparticles endows them with the ability to deliver current antifungal agents by various routes of administration, such as oral, nasal, and intraocular routes [117].

## 4. Nanotechnology-Based Therapies for Fungal Infections

Since nano theory was firstly hypothesized by Richard Feynman in 1959, it has become a broad arena for integrating various areas of knowledge, such as biology, chemistry, physics, and engineering. Nanoscience has been shown to have great potential in the treatment of pathologies [118]. Moreover, nano-sized carriers enable the delivery of multiple drugs or imaging agents in the treatment of cancer or infections and in pathologic diagnostics [119,120]. The advantages of using nano-sized carriers include prolonged drug release, resistance to metabolic degradation, augmented therapeutic effects, and even avoidance of drug resistance mechanisms [119]. Metallic nanoparticles, mesoporous silica nanoparticles, polymeric nanoparticles, and lipid-based nanosystems are possible solutions to the challenges faced in the treatment of fungal infections. As the threat of invasive and superficial fungal infections continuously increases, hundreds of studies have led to a variety of synthesized and fabricated nanosystems for the optimization of antifungal therapy. 

## 5. Metallic Nanoparticles

Metal nanoparticles are 1 to 100 nm in size and offer advantages of chemical stability, potential antifungal effects, low toxicity, and low pathogen resistance [121,122,123,124]. They can inhibit fungal cell membrane synthesis and certain fungal protein syntheses, as well as facilitate the production of fungal reactive oxygen species [125,126,127,128]. Gold, silver, zinc, and iron oxide nanoparticles are the most studied for antifungal drug delivery [121]. Several related studies are listed Table 3.

Nano-sized gold materials have been shown to have anti-candida effects with low toxicity [129,130]. Normally, gold nanoparticles are conjugated with effective agents to improve their antifungal effects. For example, indolicidin, a host defense peptide, was conjugated with gold nanoparticles to treat fluconazole-resistant clinical isolates of *C. albicans*. The indolicidin-gold nanoparticles did not show cytotoxicity for the fibroblast cells and erythrocytes and they significantly reduced the expression levels of the *ERG11* gene in *C. albicans* [130]. 

Other methods of obtaining antifungal nanoparticles include the SnCl_2_ and NaBH_4_ based synthesis methods, which provide nanoparticles average sizes of 15 nm and 7 nm, respectively. Interestingly, the smaller size of gold nanoparticles displayed better antifungal activity and greater biocidal action against *Candida* isolates than 15 nm gold nanoparticles by restricting the transmembrane H^+^ efflux [131]. In another study, triangular gold nanoparticles were synthesized and conjugated with specific peptide ligands that inhibit secreted aspartyl proteinase 2 (Sap2) in *C. albicans*. Both non-conjugated and peptide gold nanoparticles showed high antifungal activity for 30 clinical isolates of *C. albicans*, although the peptide-conjugated nanoparticles had the highest uptake efficiency [129].

Silver nanoparticles have been shown to have great potential for antifungal growth and avoiding resistance in microorganisms [132]. As with gold, silver nanoparticles are easily modified and synthesized and display stable physicochemical characteristics [133]. Monotherapy with silver nanoparticles has been evaluated in various studies in vitro, where the growth and survival of *C. albicans* and *C. tropicalis* were significantly hampered. Moreover, they show great potential against fluconazole-resistant isolates of *C. tropicalis* in clinical settings. The antifungal efficiency of silver nanoparticles can be optimized when used in conjugation with AmB and fluconazole [134,135,136]. 

Silver and gold nanoparticles have also been biosynthesized to fight fungi-induced dermal infections. Interestingly, the growth of *Candida, Microsporum,* and *Trichophyton* dermatophyte isolates was inhibited by silver particles, but *C. neoformans* was susceptible to both gold and silver nanoparticles. Both of these heavy-metal-based nanoparticles were shown to lack cytotoxicity to human keratinocytes [137]. Despite its ability to impart anti-fungal activity, an overload of silver is toxic to mammalian cells, so the toxicity and use of silver nanoparticles needs further evaluation.

Aside from directly inhibiting the growth of fungal pathogens, a low dosage of silver nanoparticles has been demonstrated to have great potential for inhibiting mycotoxin biosynthesis [138]. Mycotoxin contamination has affected over 25% of the world’s crops and leads to losses of around 1 billion metric tons of foods and food products annually according to the Food and Agriculture Organization of the United States. *F. chlamydosporum* and *P. chrysogenum* were used to produce biogenic silver nanoparticles, which inhibited the fungal growth of *A. flavus* and completely prevented its aflatoxin production [139]. *A. terreus* and *P. expansum* were also used to produce silver nanoparticles, which inhibited *A. orchraceus* and its mycotoxin production [140]. The uptake of these silver nanoparticles is believed to be localized to the endosomes. They are thought to significantly influence the fungal cells’ oxidative stress response and secondary metabolism, as well as to increase transcripts of the superoxide dismutase, which is associated with aflatoxin inhibition [138].

Zinc-containing metallic nanoparticles are also commonly studied. Zinc oxide nanoparticles are considered the most promising of these for drug release and low toxicity [141,142,143]. As with silver nanoparticles, zinc nanoparticles show significant anti-candida effects both as a monotherapy [144,145] and in combination with antifungal drugs such as fluconazole [146]. Thus far, the in vitro antifungal activities of zinc nanoparticles have been evaluated with various strains of *C. albicans, C. krusei, C. aprapsilosis,* and *C. tropicalis* [116,144,147]. However, the in vivo studies remain unconvincing; as a result, zinc nanoparticles are currently not indicated for the treatment of a specific candidiasis. Biomedical applications of iron oxide nanoparticles have also been widely investigated due to several attractive characteristics, including magnetism, biocompatibility, and stability [148,149]. Although this type of nanoparticle is mainly used in tissue imaging to assist the diagnosis, several studies indicate its great potential in treating antifungal infection. For example, *Candida* species are able to form a drug-resistant biofilm in medical apparatuses and instruments, such as catheters. Thus, Chifiriuc et al. synthesized oleic acid and CHCl_3_ fabricated iron oxide nanoparticles (Fe_3_O_4_/oleic acid: CHCl_3_) as a delivery system to carry essential oil from *Rosmarinus officinalis* and cover the catheter pieces. According to confocal laser scanning microscopy, they found that the essential oil and pulsed iron oxide nanoparticles significantly inhibited the fungal adherence of *C. albicans* and *C. tropicalis.* Furthermore, the same research group investigated these nanoparticles for their anti-bacterial capabilities by inhibiting the biofilm formation of *Enterococcus faecalis* [150,151]. 

Aside from anti-fungal effects, metallic nanoparticles have been used in fungal diagnoses [152]. The two common causes of human cryptococcosis, *C. neoformans* and *C. gatti,* have distinct pathogenic properties, so they require different therapeutic strategies. Detecting *Cryptococcus* in clinical specimens is time-consuming, and diagnosis is difficult. Artificial positively charged silver nanoparticles have been evaluated to directly distinguish between *C. neoformans* and *C. gattii* in clinical specimens using surface-enhanced Raman scattering and spectral analysis. These nanoparticles resulted in better signals than the standard substrate of negatively charged silver nanoparticles in that they self-assembled on the surface of the cryptococcal cell walls via electrostatic aggregation. This novel method based on silver nanoparticles was 100% accurate in distinguishing between the two *Cryptococcus* species.

## 6. Mesoporous Silica Nanoparticles

Mesoporous silica nanoparticles (MSNs) were firstly developed in the early 1990s and have been shown to have potential applications in areas of biomedicine, such as drug delivery, disease diagnosis, medical imaging, and tissue regeneration [155,156,157]. MSNs are fascinating in drug delivery because they are biocompatible, they are highly chemically and thermally stable, and they provide a large surface area for carrying drug payloads. Moreover, bioactive components can be assembled on the nanoparticle surface and guide the drug-loaded system directly to a specific location [158]. Thus, MSNs have been developed as novel drug delivery systems in the treatments of various diseases [158,159,160]. Table 4 shows a summary of several successful studies that use this nanomaterial in drug delivery systems for treating fungal infections.

pH-sensitive gated MSNs were synthesized for carrying tebuconazole. The assembled nanosystem significantly inhibited yeast growth and could possibly provide an optimal treatment for vaginal mycoses [161]. Furthermore, econazole incorporated into an MSN system has been demonstrated to have antifungal activity against *C. albicans* [162]. Aside from treatment strategies, MSN systems have also been used to study the functionality of macrophages in fungal infections. Portoles et al. developed mesoporous SiO_2_-CaO nanospheres that were labeled with fluorescein isothiocyanate (FITC-NanoMBGs), and the red fluorescence signal from this fluorescent-labeled MSN system was used to measure the ability of *C. albicans* to carry out phagocytosis. This provides a critical method for evaluating the macrophage-fungus interface and offers a perspective for understanding host immunity responses during fungal infections [164].

The controlled release of a drug is considered one of the most attractive improvements in anti-fungi therapeutic strategies. Polylactic acid was coated with functionalized MSNs, and this nanosystem was conjugated with levofloxacin to form an MSN-based drug delivery system. In vitro antimicrobial tests showed that this MSN-based nanosystem had significant anti-microbial properties against *Staphylococcus aureus*, *E. coli,* and *C. albicans*. However, this nanosystem was not effective in preventing the growth of *A. niger*. Nevertheless, this nanosystem was shown to have acceptable cytotoxicity to normal human fibroblasts [163].

MSNs have also been used in combination with metallic nanoparticles, which enhance the fungicidal effects. In 2016, a laboratory from Spain developed a nanosystem that utilized electrospun cellulose acetate to contain silver and copper nanoparticles and supported it with sepiolite and mesoporous silica. This resulted in significantly fungistatic membranes against *Aspergillus niger* [165]. The controlled release of metal was facilitated by the MSNs. Thus, the antifungal effects were optimized by the synergic use of metallic nanoparticles and MSNs.

Although the cytotoxicity of MSN-based antifungal treatments is acceptable [161,163], overloads of antifungal drugs or heavy metals lead to severe side effects. Thus, the use of naturally isolated antimicrobial agents might be the optimum antifungal therapy. Klouček’s research group loaded an essential oil component, eugenol, into MSNs and capped them with various saccharide gates, such as starch, maltodextrin, maltose, and glucose. In vitro experiments showed that the eugenol-loaded MSN system inhibited the growth of *A. niger* and that the release of eugenol could occur through the degradation of the anchored saccharides by exogenous enzymes.

## 7. Polymeric Nanoparticles

Polymeric nanoparticles (PNs) were first developed in the 1970s, and range in size from 5 to 1000 nm. Active ingredients such as antifungal drugs can be loaded by either entrapping them inside the particle or adsorbing them onto polymeric cores. Polymeric nanoparticles are divided into two classes, based on their morphological properties: nanocapsules and nanospheres [166]. A nanocapsule is covered by a polymeric wall and has oil or water in the core, whereas a nanosphere is made up of a continuous polymer network that can retain a drug inside or absorb it onto the surface [166,167,168]. Thus, nanocapsules and nanospheres can be considered reservoir systems and matrix systems, respectively. Several production methods have been used, including polyelectrolytic complexation, ionotropic gelation, and emulsification by evaporation, solvent diffusion, solvent evaporation, or nanoprecipitation [169,170,171,172].

The efficiency of drug dispersion into PNs is determined by several factors, including the physicochemical characteristics of the drug, nanoparticle surface characteristics, the nature of the polymer, and the amount of drug to be loaded. Thus, natural oils and polymeric surface surfactants are used to improve the drug payload in PNs [173,174]. The advantages of PN-based treatments include enhanced therapeutic efficiency and penetration, reduced toxicity, and the delivery of drug action to a target site [175]. This nanosystem has been investigated as an effective therapeutic strategy for treating fungal infections, typically with drugs that are already in use (Table 5).

To improve the therapeutic effects of antifungal agents, PNs have been widely used in the treatment of candidiasis, including oral candidiasis [176,177,186] and vaginal candidiasis [178,179,180,181,182]. In the former, chitosan was incorporated into PNs, resulting in inhibition of the biofilm adhesion and formation of *Candida*. Chitosan-PNs significantly inhibited *Candida* biofilm formation and decreased the number of colony forming units of *Candida* spp., in contrast with NaOCl, which was used as a positive control [177]. 

In other cases, PNs were used to optimize antifungal therapies by taking advantage of the large contact surface of the vagina, on which local or systemic fungal infections are obtained. Because it has a broad spectrum of action, itraconazole is a common treatment for vaginal fungal infections. In an attempt to optimize itraconazole treatment, the drug was loaded onto both nanocapsules and nanospheres, which improved the encapsulation efficiency. In a mouse model of vaginal *Candida* infection, itraconazole-loaded nanocapsules significantly reduced fungal infection, whereas loaded nanospheres did not. Histological analysis indicated that mice treated with the loaded nanospheres had augmented levels of IL-1β and TNF-α and typical tissue inflammation. This showed that the nanocapsules were a more ideal means of improving vulvovaginal candidiasis and a host of immune responses [178]. 

Another common antifungal drug used for vaginal candidiasis is AmB. In one recent study, nanoprecipitation was used to load AmB onto Eudragit RL100 nanoparticles coated with hyaluronic acid. The Eudragit RL100 nanoparticles facilitated 81% AmB release that lasted for 96 h, although the vaginal fungal burden was eliminated within 24 h. Thus, this PN system provided an optimized drug release platform that significantly promotes antifungal effects. Moreover, this study suggested that the innovative PNs facilitate AmB penetration into the vaginal epithelium via CD44 receptors, rendering this treatment a great improvement over conventional PNs and thus representing a novel vaginal means of optimizing vulvovaginal candidiasis treatment [181].

Polymeric nanoparticles have also displayed low toxicity in murine fibroblasts [180], human keratinocytes [187], kidney epithelial cells, and murine macrophages [183]. Consequently, they can provide nanosystems for optimized treatment of candidiasis. *Cryptococcus* infections have similarly been treated using PNs. In one case, AmB was loaded onto polybutylcyanoacrylate nanoparticles, which have a great ability to cross the blood-brain barrier (BBB), resulting in an effective treatment for cryptococcal meningoencephalitis. The loaded nanoparticles significantly increased the survival rate of BALB/c mice with cryptococcal infections, and the number of colony-forming units was much lower in the brain tissues of mice treated with nanoparticles compared to those treated with AmB alone. Thus, PNs have great potential for delivering antifungal drugs across the BBB as treatments for encranial infections [184]. 

The pulmonary region is another infection site for cryptococcosis, where the pulmonary mucus layer is the first point of contact with *C. neoformans*. In order to eliminate Cryptococcal related pulmonary infection, Li and Liao’s team developed a sophisticated drug carrier based on chitosan-conjugated poly (lactic-*co*-glycolic acid, PLGA) nanoparticles [185]. Chitosan is not only part of the ectocellular structure of *Cryptococcus neoformans*, but also a typical biomaterial for improving drug oral absorption. Thus, they firstly utilized the phage display library to select a chitosan-binding peptide (12-mer peptide: ADGVGDAESRTR, CP) that covalently conjugates to PLGA to form CP-PLGA NPs. This was followed by incubation with free chitosan in vitro to form chitosan covered nanoparticles, which were referred to as C-CP-PLGA NPs. 

The outer and noncovalent chitosan is responsible for enhancing the CP-PLGA NPs’ permeability across the oral absorption barrier. Furthermore, bound chitosan enhances the adhesion to the mucosal layer. The CP-PLGA NPs can be released into circulation to research an infection site. After loading the itraconazole (ITZ) into PLGA, both C-PLGA/ITZ and C-CP-PLGA/ITZ showed remarkable clearing capability for cryptococcal lung infections and increased the survival rate of mice. However, the mortality of C-CP-PLGA/ITZ treated cryptococcsis mice was significantly lower than that of C-PLGA/ITZ treated mice [185].

## 8. Lipid Based Nanoparticles

**Figure 2:**
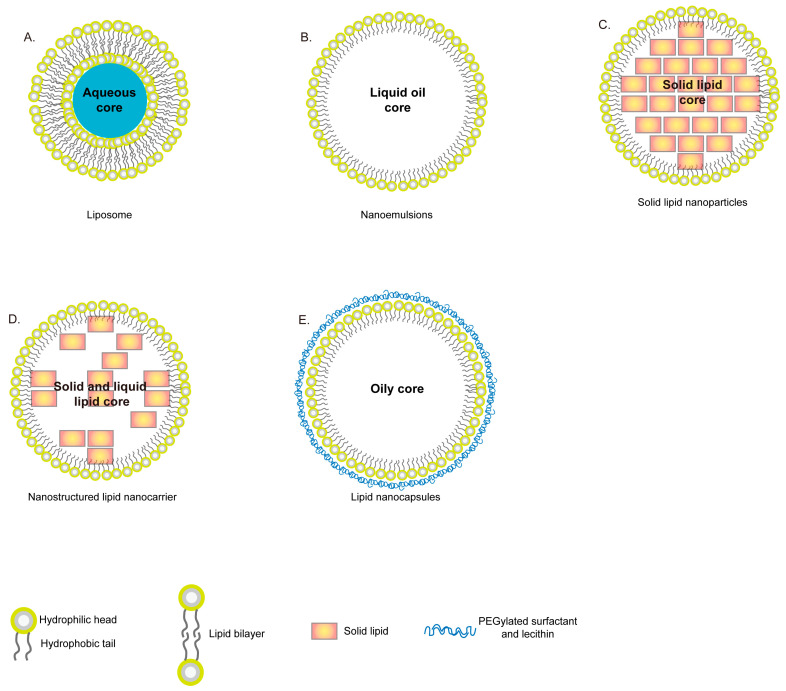
Schematic representation of lipid-based nanosystems. (**A**) Liposomes are developed as double-layer lipid systems that consist of phospholipid layers and aqueous core. (**B**) Nanoemulsions (NEs) are dispersed nanosystems that consist of a single layer of phospholipid and two types of core, either oil-in-water or water-in-oil. This system is always stabilized with an appropriate surfactant. The size of NEs is typically smaller than 500 nm. (**C**) Solid Lipid Nanoparticle (SLNs) with submicron sizes that range from 50 to 100 nm. SLNs are similar to NEs, but SLNs contain the solid lipid core that offers the enhanced drug release control and system stability. (**D**) Nanostructured Lipid Carriers (NLCs) are considered the second generation of SLN, which contain a hydrophobic nucleus dispersed in an aqueous environment and stabilized by surfactant molecules. It contains both a liquid and solid lipid core that facilitate drug release control and reduce drug expulsion during development and storage. (**E**) Lipid Nanocapsules (LNCs) were the newest developed particles ranging from 20 to 100 nm, and consist of an oily nucleus of medium-chain of triglycerides and are surrounded by a shell of PEGylated surfactant and lecithin.

Lipid-based nanosystems are also excellent drug delivery tools for treating fungal infections, reaching therapeutic targets, and providing effective delivery systems (Figure 2).

Liposomes were firstly developed as double-layer lipid systems for drug delivery in 1965. Phospholipids are the primary chemical component of the liposomal structure and offer both hydrophobic and hydrophilic characteristics. Artificial liposomal structures are primarily cholesterol, which provides advantages such as promising stability, moderation of the fluidity of the lipid bilayer, controlled drug release, and stabilization of the vesicles [188,189,190]. These constituents give liposomal structures the ability to incorporate drugs of various polarities: hydrophilic substances are encapsulated in the aqueous region, but hydrophobic substances are held inside the liposome [191].

Liposome nanoparticles are easily fabricated and modified to improve the therapeutic effects of antifungal drugs. The advantages of liposomal nanoparticles also include good biocompatibility and biodegradability. Additionally, they have considerable therapeutic potential and have desirable pharmacokinetics and pharmacodynamics, providing sustained drug release and reduced toxicity [192]. As a result, liposomes are widely utilized as primary delivery vehicles for pharmaceuticals [193,194,195]. Table 6 showed several studies that utilized liposome nanoparticles for treating fungal infections.

Fungal infections are treated using AmB, the “gold standard” among polyene-based antifungal drugs. Because it is relatively hydrophobic, conventional administration relies on intravenous administration. However, it is significantly nephrotoxic and is associated with infusion-related reactions (up to 53% of patients experience renal dysfunction) [225]. The AmB-liposome conjugates AmBisome^®^ (a liposomal amphotericin) and Abelcet (an AmB-lipid complex; The Liposome Co., Princeton, NJ, USA) were developed as alternatives to AmB deoxycholate injections. Both are successful commercial nanoformulations of this antifungal drug. Liposomal AmB agents were developed to overcome the severe side effects and nephrotoxicity of AmB [226], and AmB lipid complexes have been developed over decades. Amphotec, an AmB colloidal dispersion system, was also developed. It offers lower renal toxicity and lower mortality according to a case study of 174 patients with invasive aspergillosis [196]. In another case study of 556 patients with various invasive fungal infections, Albecet showed anti-aspergillosis effects with moderately compromised nephrotoxicity compared to AmB [197]. Albecet appears to be better at treating filamentous fungal infections (*Aspergillus*) than yeast infections (*Candida, Cryptococcus*).

AmBisome^®^ provides much greater antifungal effects against *Candida* [227]. Aside from being a liposome-based treatment for *Candida*, this nanosystem has been used to optimize *Cryptococcus* treatment strategies, although the literature is limited. Fu et al. firstly selected a fluorescent polypyridyl ruthenium complex, RC-7, with significantly lower minimum inhibitory and minimum fungicidal concentrations than fluconazole. It was loaded into brain-targeting nano-liposome particles to form RDP-liposome (RDP is a peptide derived from rabies virus glycoproteins). As a result, the fluconazole loaded RC-7 liposome nanoparticles could be guided through the BBB and accumulate in the brain. Remarkably, RDP-liposome prolonged the survival of meningoencephalitis-bearing mice [198]. Despite the many advantages of using liposomes for delivering antifungal drugs, their disadvantages cannot be overlooked. Liposome nanoparticles are soluble, they have short half-lives, and they oxidate and hydrolyze phospholipids [121].

Nanoemulsions (NEs) are dispersed systems of two immiscible liquids (either oil-in-water or water-in-oil) that are stabilized with an appropriate surfactant [228,229]. Particles of these NEs are typically smaller than 500 nm, and they have good stability, rapid digestibility, controlled release, and good bioavailability. Moreover, their physicochemical characteristics allow the delivery of various drugs. The oily component of NEs varies (e.g., essential oils or triglycerides) based on their distinct physicochemical and biological properties, and the water layer can be one of various aqueous components. For instance, because essential oils have been shown to have antifungal effects, they can be used in the oily component [230,231]. 

One NE containing essential oil from *Pelargonium graveolens* displayed anti-candida activity. *Candida albicans* and *C. krusey* showed less biofilm formation using this NE compared to the free essential oil [199]. *Ocimum basilicum* essential oil loaded in an NE showed augmented activity against *C. albicans* and *C. tropicalis* [231]. Two additional natural essential oils from *Pogostemon heyneanus* and *Eucalyptus globulus* were isolated and loaded into NEs, resulting in excellent inhibitory effects on both yeast growth and biofilm formation [200,201]. 

Even when plant-isolated essential oils are used, NEs can provide ideal delivery agents for known antifungal drugs. A nystatin-loaded NE has been tested against *C. albicans* for the treatment of skin candidiasis. Results have shown improved pharmacokinetic release of nystatin with good sustainability in the skin and NE acts as an optimal agent for treating skin candidiasis [202,203]. Additionally, ketoconazole and clove oil loaded into an NE showed strong disturbance of *Candida* fungal cell membranes and a better ketoconazole release profile compared to ketoconazole alone [204]. 

Besides conventional NEs in antifungal applications, gelling agent and thioglycolic acid have been used as permeation enhancers to improve ketoconazole’s effect on onychomycosis. The optimized NE is referred to as nanoemulgel [206]. Nanoemulgel displayed 77.54 ± 2.88% transungual permeation values for ketoconazole through goat hooves in 24 h, in contrast to conventional NEs without a permeation enhancer (transungual permeation values = 62.49 ± 2.98%). Thus, there is no doubt that ketoconazole loaded nanoemulgel shows a significant zone of inhibitionfor *T. rubrum* and *C. albicans*. Nanoemulgel also has good biosafety according to skin irritation and histopathology studies on rat skin. 

To improve the antifungal activities against *C. albicans* and *A. niger* for topical delivery, AmB-loaded NE was fabricated using Capmul PG8 and lipid surfactants. AmB-loaded NE had zones of inhibition of 19.1 ± 1.4 and 22.6 ± 2.0 mm against *A. niger* and *C. albicans*, respectively. Ex vitro experiments demonstrated that this nanosystem enhances AmB release and has the highest rate in penetrating the stratum corneum barrier compared to AmB drug solution and Fungisome^®^ (Liposomal AmB, commercial drug) [205]. In another system, NE provided an optimized nanosystem for improving AmB’s local antifungal effect without theoretical systemic absorption [232]. Drug-loaded NEs have been demonstrated to have great potential therapeutic roles for topical fungal infection, but their antifungal roles in the treatment of invasive fungal infections need further evaluation. 

Solid lipid nanoparticles (SLNs) are similar to NEs, but the essential oil component is replaced by solid lipids to improve the control of drug release, increase system stability, and reduce toxicity [233]. They are colloidal systems of submicron size, ranging from 50 to 100 nm [234,235]. The concentration of the solid lipid component ranges from 1% to 30% *w*/*w*, whereas that of the surfactants ranges from 0.5 to 5% [236]. Because the solid lipid core is lipophilic, several hydrophobic drugs can be packed into this nanosystem. Antifungal drugs such as miconazole, AmB, fluconazole, ketoconazole, and voriconazole have been carried by SLNs.

Nokhodchi et al. developed fluconazole-loaded SLNs and demonstrated its significant inhibition of fluconazole-resistant strains of *Candida* [207]. After measuring the antifungal activities, fluconazole was released dramatically from SLNs in the first 30 min followed by a sustained release over 24 h. The MIC_50_ of fluconazole-loaded SLNs for fluconazole-resistant strains were evaluated (2 μg/mL, 1 μg/mL and 2 μg/mL for fluconazole-resistant strains of *Candida albicans, Candida parapsilosis* and *Candida glabrata*, respectively) This nanosystem based drug delivery system for inhibiting *Candida* strains exhibited low susceptibility against the conventional formulation of fluconazole. 

Miconazole is another common broad-spectrum drug used against various fungal species, but it has poor water solubility. The use of SLNs alleviates this problem. Thus, various approaches have been used to enhance the antifungal efficiency of miconazone, such as adding surfactants or incorporating lipid nanoparticles, including SLNs [208,237,238], the latter of which also improves biodegradability, biocompatibility, and protection of the incorporated drug from chemical degradation [239]. In cutaneous and vulvovaginal candidiasis, ketoconazole dispersed into SLNs showed anti-candida activity [209,210]. Voriconazole, another common anti-fungal drug, was loaded into SLNs, and two studies showed antimycotic activity against *C. glabrata* when administered ocularly [211,240]. 

Besides azole drugs, as the antifungal gold standard, AmB has also been utilized as an effective agent for incorporation into SLNs to ameliorate its poor oral bioavailability. Both in vivo and in vitro pharmacodynamic and pharmacokinetic studies show that AmB loaded into SLNs provides better bioavailability and lower nephrotoxicity, and the anti-candida efficiency of AmB appeared to be potentiated [212,213,241]. To optimize cutaneous treatments, terbinafine hydrochloride was incorporated into SLNs, resulting in prolonged drug activity and a promising alternative for the treatment of cutaneous candidiasis [213]. 

Not only can SLNs incorporate single antifungal drugs, they can take on and deliver a combination of antifungal agents. A drug delivery system that simultaneously transports two or more drugs to a target site represents a promising strategy that can improve the therapeutic effectiveness of those drugs and overcome drug resistance. Clotrimazole and alphalipolic acid were loaded into SLN, and various effects were found, including reductions in reactive oxygen species and allergic responses. In vitro, the combination showed prolonged drug release without a burst effect. In total, the growth of 25 strains of *C. albicans* was inhibited by this dual-drug delivery system. This study demonstrated that the antifungal effect of clotrimazole and the protective effect of alphalipolic acid could be combined using this optimized strategy for the treatment of candidiasis [214]. 

Finally, SLCs have been studied as a means of delivering natamycin with improved therapeutic effects against fungal keratitis [215]. This lipid-based drug delivery system showed sustained natamycin release and better corneal penetration compared to the drug alone. The SLCs significantly improved the antifungal effectiveness of natamycin against clinical isolates of *A. fumigatus* and *C. albicans* without cytotoxic effects on corneal tissues. With this evidence, the properties of SLNs render them the ability to significantly increase the bioavailability of antifungal agents while alleviating their side effects. Such SLN nanosystems are favorable for the treatment of cutaneous, oral, ocular, and vaginal fungal infections.

A nanostructured lipid nanocarrier (NLC) is a nanoscale colloidal system that has a hydrophobic nucleus dispersed in an aqueous membrane and is stabilized by a surfactant. It is considered an improvement over SLNs, although the manufacturing methods are similar [242,243]. The attractive properties of a synergic NLC system include nanoparticle size (<100 nm), broad size distribution, long-term stability, and low cytotoxicity to human cell lines (HaCaT and A431). The hydrophobic nucleus gives an NLC better biocompatibility and a larger drug payload compared to the solid lipids in SLNs. Moreover, substitution of the nucleus can improve drug release properties and the incidence of drug expulsion during development and storage [236,244,245]. Thus, broad-spectrum drugs carried by NLCs have applications in the treatment of fungal infections. 

Several azole drugs with poor water solubility (voriconazole, clotrimazole, miconazole, fluconazole, and sertaconazole) have been incorporated into NLCs and used to treat fungal infections. One study indicated that voriconazole loaded onto NLCs significantly alleviated biofilm formation and fungal growth of *C. albicans.* This shows the critical role of NLCs in facilitating the inhibition effects of voriconazole against yeast–hyphae switching, a known critical virulence characteristic of the *Candida* species [216]. 

In another study, fluconazole-loaded NLCs were prepared using probe ultrasonication. The particles were approximately 126 nm in diameter and provided sustained drug release over 24 h, resulting in a significant decrease in minimum inhibitory concentration for all *Candida* species. In particular, isolates of *C. albicans* were more susceptible to fluconazole-loaded NLCs than *C. glabrata* or *C. parapsilosis* [217]. 

To optimize the treatment of vaginal fungal infections, clotrimazole was dispersed into NLCs, improving its anti-fungal effects by about four times compared to commercial Fungizone^TM^ [218]. The NLCs produced by ultrasonication were viscosized by the addition of poloxamer P407 gel to obtain a clotrimazole-NLC-gel nanosystem. The most significant advantage of this system is its thermogelling property, by which the temperature of vaginal fluids is increased by the clotrimazole-NLC-gel. This facilitates the ability of clotrimazole to pass through the vaginal mucosa, resulting in improved antifungal effectiveness in the treatment of vaginal candidiasis. 

To optimize treatments of oral fungal infections, miconazole has been used as the loaded drug, and this NLC-based hydrogel system controlled the release of miconazole [219]. The encapsulation of miconazole into NLCs improved its antifungal activity against *C. albicans*, giving this nanosystem the ability to obtain the same therapeutic effect with a 17-fold lower dose than a commercial oral gel formulation. 

The therapeutic effects of itraconazole against dermal candidiasis have also improved with the use of NLCs [220]. Itraconazole-loaded NLCs decreased trans-epidermal water loss, an index of cutaneous barrier function, in intact skin and tissues damaged by a linear incision. This indicates that the NLCs improved the ability of itraconazole to localize within the skin. This NLC system was evaluated only on *Galleria mellonella* larvae, and in that setting, it showed significant antifungal effects against *Sporothrix brasiliensis* and *C. albicans* [220]. 

In another study, an NLC-based nose-to-brain drug delivery system was developed to overcome the poor ability of ketoconazole to penetrate the BBB. A large amount of fluorescent-labelled NLCs accumulated in the brain, and ketoconazole-loaded NLCs significantly inhibited the number of colony-forming units of *C. neoformans* in mouse brains [221]. The use of NLCs is not limited to delivering single antifungal drugs. A Mediterranean essential oil (*Rosmarinus officinalis, Lavandula* x *intermedia,* or *Origanum vulgare*) was loaded along with clorimazole in an NLC system, which increased the drug’s effectiveness in an optimized treatment for *Candida* infections [222]. The essential oil enhanced the dermal permeability of the clotrimazole, possibly as a result of its excellent interaction with the skin, and the nanoparticles provided prolonged in vitro release of clotrimazole. The abilities of *C. albicans, C. krusei,* and *C. parapsilosis* to grow were significantly hampered, thus confirming that NLCs containing Mediterranean essential oils and clotrimazole represent a promising strategy for enhancing drug effectiveness against topical candidiasis. 

The antifungal gold standard drug, AmB, has been used for many years. To optimize its effectiveness in the treatment of ocular fungal infections, AmB was loaded into a polyethylene glycol PEGylated NLC system using a high-pressure homogenization technique. The molecular weight of the PEG was varied to find the one that provided the best stability. When the NLC was PEGylated with 2K PEG, the system remained stable for at least 4 weeks at both 4 °C and 25 °C. In vitro, this system provided excellent antifungal activity against *C. albicans* and *A. fumigatus*. Compared to commercially available AmB formulations such as Fungizone^TM^ and AmBisome^®^, it showed significantly better antifungal activity, and compared to AmB alone, it showed lower cytotoxicity. 

This structure facilitated the ocular bio-distribution of AmB when applied topically in animal experiments. The fabricated NLC system has an excellent ability to sustain antifungal agents, control drug release, and reduce *Candida* activity in ocular regions in animals. This optimized method can provide an innovative strategy for treating various fungal infections.

Compared to SLNs, most NLC-based drug delivery systems can be used to combat invasive and systemic fungal infections as well as those of a topical nature. They can also restrict fungal biofilm formation. In summary, this nanosystem provides a promising method for optimizing current antifungal therapies, given their unique properties of high biocompatibility, optimized bioavailability, and varied routes of administration.

Lipid nanocapsules (LNCs) are lipid-based nanocarriers that have a relatively novel structure, given that they have recently been introduced (in the 2000s). They consist of a core containing medium-chain triglycerides that is stealth-coated with a relatively rigid phospholipid/ethoxylated surfactant capsule [246]. Unlike SLNs and NLCs, LNCs are prepared using a low-energy phase-inversion method. The nanocapsule is less than 100 nm in size and has a narrow size distribution. It has high interfacial stability and excellent stability with the payload [247,248]. Such nanosystems have been developed as an effective cancer drug delivery tool to be used via parenteral [249], topical [250], or oral routes of administration [251,252]. 

Promising outcomes have been achieved when using LNCs for the delivery of antifungal drugs by various routes of administration. To optimize the treatment of dermal candidiasis, itraconazole-loaded LNCs were compared to itraconazole-loaded NLCs. The lipid nanocapsules showed significantly better in vitro characteristics such as the polydispersity index, itraconazole release profile, and antifungal activity against *C. albicans*. When applied topically, the nanocapsules showed better dermal retention of itraconazole in excised human skin. They provided a critical therapy for the treatment of induced cutaneous candidiasis in rats while avoiding histopathological changes in the epidermal and dermal layers of the skin during a 14-day treatment. 

The study showed that LNCs have superior physicochemical characteristics for permeating the skin and treating dermal candidiasis. Resistance is another major problem with azole treatments. To evaluate fluconazole resistance reversion in *C. albicans, C. glabrata, C. krusei,* and *C. tropicalis* isolates, LNCs with modified lipid cores were loaded with fluconazole. The LNCs provided advantages such as a reduced fluconazole dose and resistance reversion in all *Candida* isolates. This study provided a potential application for an azole-LNC nanosystem in the treatment of fungal infections caused by resistant isolates of *Candida* [223].

To treat cryptococcosis, LNCs have been evaluated for their anti-cryptococcal effects in murine models [224]. This novel study focused not only on the use of LNCs in the delivery of conventional azole drugs, but also on developing synergic drugs for treating cryptococcosis in mice. The target was a fungal calcium channel and a selected blocker, amiodarone, was used as an alternative therapy. Amiodarone is an antiarrhythmic drug that shows broad-spectrum antifungal effects and is effective against *C. neoformans* [253]. It can interrupt the calcium-calcineurin signaling pathway, significantly compromising cryptococcal virulence [254,255]. It has also been used to treat fluconazole-resistant fungi [256]. 

In a recent study, amiodarone and fluconazole were loaded singularly and in combination into LNCs. Each one showed antifungal effects when administered intranasally, but the LNCs improved the anti-cryptococcal effects for amiodarone only. Therefore, the LNC system is a critical strategy for improving drug loading that improves efficiency and optimizes conventional cryptococcosis treatments.

Hydrophobic agents such as AmB and azoles can be dispersed into any of the lipid-based nanosystems: SLNs, NLCs, and LNCs. All of these are used in attempts to circumvent adverse effects and improve bioavailability in the treatment of oral, ocular, vaginal, or dermal fungal infections. Moreover, synergic incorporation of antifungal agents with natural biomolecules, such as plant-isolated essential oils, allows lipid-based nanosystems to boost drug sustainability, prolong drug release, and improve antifungal effects [224]. Thus, these three lipid-based nanosystems provide promising methods for optimizing antifungal therapies. However, in vivo experiments must be evaluated further.

## 9. Conclusions and Perspectives

Fungal infections have become a global burden and cause significant mortality. Conventional antifungal drugs have unsatisfactory therapeutic benefits, severe side effects, or unfavorable physicochemical properties. Nanoparticle-based drug delivery systems provide alternative strategies for overcoming these limitations, providing favorable properties such as small size, biocompatibility, and low toxicity. 

This review has summarized the applications of four different categories of nanoparticles in antifungal therapy, which possesses with distinct properties. The metallic nanoparticles display chemical stability, adjustable pore size, and potential antifungal activity by inhibiting fungal enzymes and inducing the production of reactive oxygen species. Currently, the antifungal effects of gold, silver, zinc oxide, and iron oxide are mainly studied among metallic nanoparticles. However, the cost of materials is a limitation for the large-scale applications of such nanosystems. 

MSNs are highly biocompatible and display satisfactory chemical and thermal stability. Due to their excellent plasticity and large drug-loading capacity, MSNs are suitable for loading large amounts of antifungal agents for treating severe fungal infection. PNs are also commonly used nanomaterials in drug-delivery systems and have been developed over half a century. Synthetic or natural polymers endow PNs with great capability to improve target guiding via surface ligand assembly, and fabricated PNs have been used as effective delivery systems for antifungal agents for decades. 

Lastly, lipid-based nanoparticles involve several variations: liposomes, NEs, SLNs, NLCs, and LNCs. Although lipids are the fundamental structural components of these nanoparticles, they have distinct properties for incorporating different antifungal agents with either hydrophobic or hydrophilic properties. Moreover, distinct lipid nanoparticles display different tissue-penetration efficiency for circumventing tissue barriers.

The purpose of developing novel delivery systems for antifungal therapies or antifungal agents is to find an effective, economical, and low-toxicity therapeutic strategy. Although these nanomaterials have distinct properties, they significantly enhance drug bioavailability and stability and display non-toxic biological characteristics that can improve the potency of antifungal drugs. Nevertheless, optimized and novel nanomaterials need to be elucidated with more animal experiments and clinical trials to evaluate their antifungal efficiency and undesirable side effects in complex environments.

## Figures and Tables

**Table 1:** Pathogenic fungi caused human diseases.

Diseases	Fungal Species	Conventional Treatments	Common Clinical Features and Symptoms
Dimorphic mycoses	*B. dermatitidis*	Azoles and polyenes	Cutaneous disease Pulmonary disease Disseminated disease
	*C. immitis*		Fever, cough, shortness of breath, chest pains Headaches, weight loss, rashes Lung cavities (commonly occurs in children)
	*C. posadasii*		
	*H. capsulatum*		Acute pulmonary histoplasmosis Chronic pulmonary histoplasmosis Disseminated histoplasmosis Histoplasmoma African histoplasmosis
	*P. brasiliensis*		Systemic mycosis, paracoccidioidomycosis
	*T. marneffei*		Common symptoms include fever, malaise, weight loss, skin and soft tissue lesions, hepatosplenomegaly, lymphadenopathy, cough and dyspnea Less common symptoms include osteoarticular involvement, abdominal pain and diarrhea [19]
Disseminated cryptococcosis	*C. neoformans*	Azoles, polyenes and antimetabolites	Cryptococcal meningocephalitis Cryptococcal pneumonia
	*C. gattii*		
Aspergillosis	*A. fumigatus*	Azoles, polyenes, echinocandins	Chronic cavitary tuberculosis Mild, self-limited hemoptysis Chronic necrotizing pulmonary aspergillosis Chronic fibrotic pulmonary aspergillosis Severe asthma Allergic bronchopulmonary aspergillosis (in atopic patients) [20]
	*A. flavus*		
	*A. terreus*		
	*A. nidulans*		
	*A. niger*		
	*A. clavatus*		
Candidiasis	*C. albicans*	Azoles, polyenes, echinocandins	Mucosal Candida infection, including oropharynx, esophagus and vagina Candidemia Acute disseminated candidiasis Infective endocarditis Vertebral osteomyelitis and diskitis Endophthalmitis Meningitis Septic arthritis Tenosynovitis [11,21]
	*C. tropicalis*		
	*C. glabrata*		
	*C. parapsilosis*		
	*C. krusei*		
	*C. auris*		
Mucormycosis	*Rhizopus* spp.	Polyenes and azoles	Tissue necrosis Sinus pain, nasal congestion, fever, soft tissue swelling and headache Blurred vision or loss of vision Cranial neuropathies or cerebral abscesses Cutaneous mucormycosis, skin swelling, necrosis and formation of abscesses [22]
	*Mucor* spp.		
	*Cunninghamella bertholletiae*		

**Table 2:** Conventional anti-fungal therapies.

Antifungal Agents	Drugs	*Targets*	Mechanisms	Administration Routes	Side Effects
Azoles	Econazole (ECO)	*Epidermophyton, Microsporum, Trichophyton*	Inhibits the fungal cytochrome P450-dependnent enzyme 14α-lanosterol demethylase encoded by the *ERG11* gene that converts lanosterol to ergosterol in the fungal cell membrane; thus, inhibits fungal growth and replication [68,69]	Topical	Well tolerated, but rare cases with local irritation, itching, and burning [70]
	Sertaconazole (SER)	*Epidermophyton, Trichophyton*			
	Miconazole (MCZ)	*Candida* spp., *Aspergillus* spp., *Cryptococcus neoformans, Histoplasma capsulatum, Pseudallescheria boydii, Trichosporon*		Topical Intravenous	May cause congenital disease when combined with metronidazole during pregnancy
	Sulconazole (SUL)	*Epidermophyton, Microsporum, Trichophyton,* *Candida* spp.		Topical	Redness, irritation, contact dermatitis, and pruritus [71]
	Tioconazole (TIO)	*Candida* spp.		Topical	Itching, discomfort, rash, erythema, mild burning, and stinging May cause severe local adverse effects [72]
	Ketoconazole (KTC)	*Blastomyces, Candida* spp., *Histoplasma capsulatum*		Topical Systemic applicatoin Oral	Hepatotoxicity and liver failure [73,74] Endocrine dysfunction, e.g., gynecomastia [75]
	Clotrimazole (CLT)	*Candida* spp., *Epidermophyton, Microsporum, Trichophyton*		Topical Oral	Gastrointestinal tract toxicity as consumption with oral lozenges Elevation of liver enzymes [76]
	Luliconazole (LUL)	*Epidermophyton, Trichophyton*		Topical	No significant side effects
	Itraconazole (ITC)	*Aspergillus* spp., *Blastomyces, Histoplasma capsulatum*		Systemic application Topical Parenteral	Diarrhea Abdominal pain Hypertriglyceridemia Pancreatitis Liver injury Cardia dysrhythmia
	Posaconazole (POS)	*Aspergillus* spp., *Candida* spp.		Systemic application	Fever, diarrhea, nausea, vomiting, headache Hypokalemia, rash Thrombocytopenia, abdominal pain Peripheral neuropathies Hepatocellular damage
	Fluconazole (FLC)	*Candida* spp., *Cryptococcus* spp.		Systemic application Topical Oral Parenteral	Liver dysfunction [77,78] Anaphylaxis
	Voriconazole (VRC)	*Aspergillus* spp., *Candida* spp., *Fusarium* spp., *Scedosporium* spp.		Systemic application	Peripheral neuropathies Pancreatitis Periostitis [79,80] Phototoxic reactions Squamous cell carcinoma
	Efinaconazole (EFI)	*Trichophyton*		Topical	No significant toxicity reported, but causes embryotoxicity in animal model [81]
	Isavuconazonium (ISA)	*Aspergillus* spp., *Mucor* spp.		Systemic application	Headache, nausea, vomiting, diarrhea, elevated liver enzymes [82]
Polyenes	Amphotericin B (AmB)	*Aspergillus* spp., *Candida* spp., *Cryptococcus* spp.	Directly interacts with cell membrane component and ergosterol, induces the formation of pores, and alters the cell permeability, causing the effusion of cytoplasmic content and fungicidal consequences	Systemic application Topical	Renal failure, electrolyte imbalance, and hepatotoxicity Fever, chills, headache, myalgias, bone marrow, and kidney toxicity
	Nystatin B (NYT)	*Candida* spp.		Oral	Mild gastrointestinal symptoms, acute renal failure [83]
	Natamycin (NAT)	*Fusarium* spp., *Aspergillus* spp. [84]	Inhibits the amino acid and glucose transportation, leads to ergosterol-specific and reversible inhibition of membrane transport proteins without altering the cell membrane permeability [85]	Topical	No severe side effects have been reported Rare cases reported mild irritation, redness, foreign body sensation, stinging, burning sensation, and tearing [86]
Echinocandins	Anidulafungin (AFG)	*Candida* spp. [87,88]	Acts as the noncompetitive inhibitor of β-1, 3-D-glucan synthase, which leads to the inhibition of the synthesis of glucan. Thus, it compromises the fungal cell wall stability and synthesis.	Intravenous	No severe side effects have been reported
	Caspofungin (CFG)	*Candida* spp., *Aspergillus* spp.		Intravenous	No severe side effects have been reported Rare cases of chills, fever, phlebitis/thrombophlebitis, tachycardia, nausea, vomiting, rash, abdominal pain, headache, and diarrhea [89]
	Micafungin (MFG)	*Candida* spp.		Intravenous	Risk of hepatocarcinogenesis Rare cases of vomiting, nausea, diarrhea [89,90]
Allylamins	Butenafine (BUT)	*Epidermophyton, Microsporum, Trichophyton* *Aspergillus* spp.	Acts as the squalene epoxidase inhibitor that inhibits the ergosterol synthesis and causes the fungal cell lysis via altering cell membrane permeability	Topical	Mild burning and/or stinging are common [91]
	Terbinafine (TRB)	*Trichophyton*		Topical	Headache Gastrointestinal symptoms Severe neutropenia Thrombocytopenia Liver failure or injury Taste, visual, and smell disturbances Depressive symptoms [92,93]
	Naftifine (NAF)	*Trichophyton*		Topical	No severe systemic side effects Local irritation and uncommon cases of allergic reaction [94]
Antimetabolites	5-flucytosine (5-FC)	*Candida* spp., *Cryptococcus* spp.	Interrupts the pyrimidine metabolism and inhibits RNA, DNA, and protein synthesis	Systemic application	Bone marrow suppression Hepatic dysfunction Diarrhea

**Table 3:** Metallic Nanoparticle based antifungal therapeutic strategies.

Nanosystems	Active Antifungal Agents	Pathogens	Target Diseases	Antifungal Mechanisms and Outcomes	References
Triangular gold nanoparticles	Antifungal peptides	Thirty clinical isolates of *C. albicans* from patients with vaginal candidiasis	Vaginal candidiasis	Antifungal effects were achieved via conjugating nanoparticles with peptide ligands that inhibit secreted aspartyl proteinase 2 (Sap2) in *C. albicans*	[129]
Gold nanoparticles	Indolicidin	Ten fluconazole-resistant clinical isolates of *C. albicans* in skin lesions	*C. albicans* caused burn infection	Conjugated indolicidin with gold nanopartilces significantly reduced the expression levels of the *ERG11* gene in fluconazole-resistant isolates of *C. albicans* and *iNOS* gene in macrophage	[130]
Gold nanoparticles	Various size of gold nanopartilces	Three clinical isolates of *C. albicans*	Anti-fungal growth	7 nm gold nanoparticles displayed higher antifungal activities than larger ones (15 nm)	[131]
Biogenic silver nanoparticles	Amphotericin B (AmB)	*C. albicans, C. tropicalis*	Anti-fungal growth	Amphotericin B-conjugated silver nanoparticles with more activity in inhibiting *C. albicans* and *C. tropicalis* as compared to AmB only	[135]
Silver/silver chloride nanopartilces	Latex of *Azadirachta indica*	Sensitive and resistant strains of *C. tropicalis*	Inhibited fungal growth and biofilm formation	Latex fabricated silver/silver chloride nanoparticles inhibited fungal growth and biofilm formation	[136]
Polyvinylpyrrolidone (PVP)-capped quantum-sized silver nanoparticles (SNPs)	Polyvinylpyrrolidone and silver	*C. albicans*	Anti-fungal growth	The MIC determined that PVP-capped SNP displayed antifungal effects in 70 ng/mL, which was lower than AmB (500 ng/mL), fluconazole (500 ng/mL), and ketoconazole (8 μg/mL)	[137]
Biogenic gold and silver nanoparticles	The high astaxanthin content yeast, *Phaffia rhodozyma,* is utilized for microbe-assisted nanoparticle synthesis	*C. albicans, C. glabrata, C. krusei, C. parapsilosis, C. tropicalis, C. neoformans, M. gypseum, T. mentagrophytes, T. tonsurans*	Treat for superficial cutaneous mycosis	Biogenic silver nanoparticles displayed significantly antifungal effects to *Cryptococcus, Candida, Microsporum,* and *Trichophyton* dermatophytes, while gold nanoparticles only showed antifungal effects to *Cryptococcus*	[138]
Biogenic silver nanoparticles by *F. chlamydosporus* (*Fusarium*-silver nanoparticles) or *P. chrysogenum* (*Penicillium*-silver nanoparticles)	Silver nanoparticles	*A. flavus, A. ochraceus*	Anti-fungal growth and inhibit aflatoxin production	The MIC results for *A. flavus* were 48, 45, and 50 μg/mL for *Fusarium* synthesizedsilver nanoparticles (FAgNPs), *Penicillium* synthesizedsilver nanoparticles (PAgNPs), and itraconazole, respectively. For *A. ochraceus*, FAgNPs, PAgNPs, and itraconazole displayed MIC values of 51, 47, and 49 μg/mL, respectively. Moreover, FAgNPs and PAgNPs completely inhibit the aflatoxin production by *A. flavus* and the MIC values were 5.9 and 5.6 μg/mL, respectively, 6.3 and 6.1 μg/mL of the *A. ochraceus* produced ochratoxin A was inhibited by FAgNPs and PAgNPs, respectively.	[139]
Zinc oxide nanoparticles	Fluconazole	*C. albicans* isolated from vaginal samples	Vulvovaginal candidiasis	Fluconazole conjugated zinc oxide nanoparticles displayed anti-candida growth effects	[146]
Zinc oxide nanoparticles	Nystatin	*C. albicans* isolated from Vulvovaginal Candidiasis	Vulvovaginal candidiasis	Nystatin conjugated zinc oxide nanoparticles anti-candida growth via inhibiting the expression of fungal *SAP1-3* genes	[147]
Oleic acid and CHCl_3_ fabricated iron oxide nanoparticles (Fe_3_O_4_/oleic acid: CHCl_3_)	*Rosmarinus officinalis* essential oil	*C. albicans* and *C. tropicalis*	Biofilm formation in the medical apparatus and instruments	The essential oil pulsed iron oxide nanoparticles significantly inhibit fungal adherence of *C. albicans* and *C. tropicalis.* Thus, they inhibit the biofilm formation in the medical instruments	[150]
Gold and silver nanoparticles	Heparin	*C. albicans, C. krusei, C. parapsilosis*	Anti-fungal growth	Silver-Heparin conjugated nanoparticles displayed antifungal effects, instead of gold-Heparin conjugated nanoparticles	[153]
Zinc oxide nanoparticles	N/A	125 clinical isolates of *C. albicans* from patients with urinary tract infections	*C. albicans* caused urinary Tract Infections	Zinc oxide nanoparticles displayed antifungal effects to 125 clinical isolated *C. albicans* strains (include 10 fluconazole-resistant strains) via inhibiting the fungal *ALS1* and *ALS3* gene expression	[154]

**Table 4:** Mesoporous Silica Nanoparticle based antifungal therapeutic strategies.

Nanosystems	Active Antifungal Agents	Pathogens	Target Diseases	Antifungal Mechanisms and Outcomes	References
pH-sensitive gated mesoporous silica nanoparticles	Tebuconazole	*S. cerevisiae*	Anti-fungal growth	Tebuconazole loaded mesoporous silica nanoparticles enable sensing of the environmental pH alteration and release the fungal agent for antifungal effects	[161]
Hexagonal mesoporous silica nanoparticle with aminopropyl groups	Econazole	*C. albicans*	Topical fungal infection	Dermal administration of econazole loaded mesoporous silica nanoparticles displayed antifungal effects to *C. albicans* in vitro and in vivo	[162]
Nanoflowers polylactic acid added with mesoporous silica nanoparticles	Levofloxacin	*S. aureus, E. coli, C. albicans, A. niger*	Anti-fungal growth	The anti-microbial effect of Levofloxacin was enhanced by functionalized mesoporous silica nanoparticles with lactic acid	[163]

**Table 5:** Polymeric Nanoparticle based antifungal therapeutic strategies.

Nanosystems	Active Antifungal Agents	Pathogens	Target Diseases	Antifungal Mechanisms and Outcomes	References
Poly-lactic acid and dextran sulfate synthesized polymeric nanoparticles	Curcumin	*C. albicans* were inoculated in mice tongues	Oral candidiasis	Polymeric nanoparticles improved the hydrophilicity of curcumin and significantly inhibited the colony-forming unit of *C. albicans* in mouse tongue tissues	[176]
Chitosan nanoparticles	N/A	*C. albicans C. tropicalis, C. krusei*	Anti-biofilm formation	Chitosan nanoparticles displayed significant fungicidal effects in *Candida* and inhibited its biofilm formation	[177]
Polycaprolactone nanoparticles with two forms: nanocapsules (NC) and nanospheres (NS)	Itraconazole	*C. albicans* were inoculated in mice vagina	Vulvovaginal candidiasis	Only itraconazole loaded NC significantly decreased fungal load in mice vaginal tissue, instead of itraconazole loaded NS	[178]
Chitosan-based polymeric nanoparticles	Miconazole	*C. albicans* were inoculated in mice vagina	Vulvovaginal candidiasis	Miconazole loaded chitosan-based polymeric nanoparticles displayed same therapeutic effects to miconazole; however, nanoparticles only encapsulated one seventh of miconazole concentration	[179]
Chitosan nanoparticles	Farnesol and miconazole	*C. albicans* were inoculated in mice vagina	Vulvovaginal candidiasis	Farnesol and miconazole loaded chitosan-nanoparticles not only inhibited fungal growth, but hampered yeast to hyphae transition	[180]
Eudragit RL100 nanoparticles coated with hyaluronic acid (EUD nanoparticles /HA)	Amphotericin B (AmB)	*C. albicans* were inoculated in mice vagina	Vulvovaginal candidiasis	AmB EUD nanoparticles/HA enable to penetrate into the vaginal epithelium via CD44 receptor and eliminated of 100% of the vaginal fungal burden within 24 h	[181]
Poly (lactide-co-glycolide) (PLGA) or poly (lactide-co-glycolide)-poly (ethylene glycol) (PLGA-PEG) blend nanoparticles	Amphotericin B (AmB)	*C. albicans, C. neoformans*	Anti-fungal growth	AmB loaded PLGA-PEG nanoparticles only displayed antifungal effects to *C. albicans*, not to *C. neoformans.* Moreover, AmB loaded PLGA-PEG not showed liver and renal damage	[182]
Chitosan nanoparticles	Bioactive peptide from Tityus stigmurus scorpion (TistH)	*C. albicans, C. parapsilosis, C. tropicalis, C. krusei*	Inhibit fungal growth and biofilm formation	TistH-Chitosan nanoparticles inhibited *C. albicans, C. parapsilosis,* and *C. tropicalis*, and also reduced the biofilm formation of *C. tropicalis, C. krusei,* and *C. albicans*	[183]
Polybutylcyanoacrylate nanoparticles (PBCA-NA)	Amphotericin B (AmB)	*C. neoformans* induced meningoencephalitis in mouse model	Cryptococcal meningoencephalitis	The intravenous administration of AmB-PBCA-NP enabled crossing the blood brain barrier and reduced the colony-forming unit counts of cryptococcal meningoencephalitis mouse model.	[184]
Poly (lactic-co-glycolic acid) nanoparticles with fungal chitosan-binding peptide	Itraconazole	*C. neoformans*	Cryptococcal pneumonia	Polymeric nanoparticle carried cryptococcal chitosan-binding peptide specific recognized and bind to fungal capsules, and thus precisely delivered the itraconazole to clear the *C. neoformans* from mice lungs	[185]

**Table 6:** Lipid Nanoparticle based antifungal therapeutic strategies.

Nanosystems	Active Antifungal Agents	Pathogens	Target Diseases	Antifungal Mechanisms and Outcomes	References
Sodium cholesteryl sulfate lipid complex with Amphotericin B, as referred to ABCD	Amphotericin B (AmB)	174 patients with invasive aspergillosis	Invasive aspergillosis	The drug-related toxicity was evaluated via comparing between ABCD and AmB recipients, 53% vs 30% (chills), 27% vs 16% (fever), 1% vs 4% (hypoxia), and 22% vs 24% (toxicity requiring study drug discontinuation). The antifungal efficiency was equivalent between ABCD and AmB	[196]
AmB lipid complex (ABLC)	Amphotericin B (AmB)	556 patients with fungal infection and intolerant of conventional antifungal therapy.	Invasive fungal infection	There were 57% of patients with responses to ABLC, including 42% (55) of 130 cases of aspergillosis, 67% (28) of 42 cases of disseminated candidiasis, 71% (17) of 24 cases of zygomycosis, and 82% (9) of 11 cases of fusariosis	[197]
Nanoliposome with RDP (peptide derived from rabies virus glycoprotein for brain-targeting)	Fluorescent polypyridyl ruthenium complex RC-7	Cryptococcal meningoencephalitis animal model with *C. neoformans* infection	Cryptococcal meningoencephalitis	RC-7-RDP nanosystem remarkably prolonged the survival days of the meningoencephalitis-bearing mice from 10 days to 24 days.	[198]
Nanoemulsions (NE)	Geranium oil (GO) from *Pelargonium graveolens*	*C. albicans, C. tropicalis, C. glabrata, C. krusei*	Inhibit fungal growth and biofilm formation	GO loaded nanoemusions inhibited growth and biofilm formation of *Candida* spp., except *C. krusei* biofilm formation with strong resistance to GO-NE	[199]
Nanoemulsions (NE)	Clove oil (CO)	*C. albicans, C. glabrata*	Anti-fungal growth	Nanoemulsions enhanced antifungal effects of CO	[200]
Nanoemulsions (NE)	Patchouli essential oil (EO) from *Pogostemon heyneanus (PH), Pogostemon plectranthoides (PP)*	Multi-drug resistant (MDR) strains of *S. aureus, S. mutans* and *C. albicans*	Anti-fungal growth	PH-EO nanoemulsion exhibited better anti-bacterial and anti-candida activities than PP-EO nanoemulsion.	[201]
Nanoemulsions (NE)	Nystatin	*C. albicans*	Buccal candidiasis	Nystatin loaded nanoemulsion displayed anti-candida effects	[202]
Nanoemulsions (NE)	Nystatin	*C. albicans, S. cerevisiae*	Skin fungal infection	Nanoemulsion sustained the release of nystatin and the antifungal drug was not absorbed into systemic circulation and sufficiently eliminated dermal *C. albicans*. Thus, this nanoemulsion system enhanced the nystatin therapy for skin candidiasis	[203]
Nanoemulsions (NE)	Ketoconazole with clove oil (CL-KTZ)	*C. albicans*	Anti-fungal growth	The fungal membrane integrity and growth were decreased in NE-CL-KTZ treated group and the nanoemulsions improved KTZ release more than nine times when compared to KTZ cream	[204]
Nanoemulsions (NE)	Amphotericin B (AmB)	*C. albicans, A. niger*	Skin candidiasis and aspergillosis	NE enhanced AmB to cross the stratum corneum barrier in rat skin and displayed better fungicidal effects than AmB and Fungisome^TM^ (commercial antifungal drug).	[205]
Nanoemulsions (NE) with thioglycolic acid as permeation enhancer	Ketoconazole	*C. albicans, T. rubrum*	Onychomycosis	Ketoconazole-NE contained permeation enhancer that displayed antifungal effects for onychomycosis	[206]
Solid lipid nanoparticles	Fluconazole	Fluconazole-resistant strains of *C. albicans*	Anti-fungal growth	Solid lipid nanoparticle promoted the fluconazole release and enhanced antifungal effects to *C. albicans, C. parapsilosis, C. glabrata*	[207]
Solid lipid nanoparticles (SLN) with hydrogel	Miconazole	*C. albicans* were inoculated into dermal side of skin in mouse model	Skin candidiasis	This SLN-hydrogel system provided sustained release of miconazole and displayed antifungal effects to *C. albicans* both in vitro and in vivo	[208]
Solid lipid nanoparticles (SLN) with hydrogel	Ketoconazole (KCZ)	*C. albicans* were inoculated to the skin in mouse model	Skin candidiasis	KCZ-SLN exhibited a sustained drug release over 24 h and inhibited topical fungal infection	[209]
Solid lipid nanoparticles (SLN) conjugated with polyoxyethylene-40 stearate (PEG-40 stearate)	Ketoconazole (KCZ) and clorimazole	*C. albicans*	Vulvovaginal candidiasis	SLN conjugated PEG-40 facilitated the stabilization of antifungal agents and enhanced fungicidal effects	[210]
Lipid-based nanoparticles (LNP)	Voriconazole	*C. glabrata, A. flavus*	Ophthalmic candidiasis and aspergillosis	LNP improved the poor solubility of voriconaole and enhanced its antifungal effects	[211]
Solid lipid nanoparticles (SLN)	Amphotericin B (AmB)	*C. albicans*	Oral candidiasis	SLN improved AmB oral bioavailability and alleviated its toxicity, and it enhanced its antifungal effects both in vivo and in vitro	[212]
Solid lipid nanoparticles (SLN)	Terbinafine hydrochloride (TH)	*C. albicans*	Skin candidiasis	SLN improved the stability and antifungal effects of TH	[213]
Solid lipid nanoparticles (SLN)	Clotrimazole (CLZ) and alphalipolic acid (ALA)	*C. albicans*	Anti-fungal growth	SLN prolonged drug release without any burst effect and facilitated antifungal effects in animal model	[214]
Solid lipid nanoparticles (SLN)	Natamycin	*C. albicans, A. fumigatus*	Fungal keratitis	SLN sustained drug release and increased corneal penetration. SLN-natamycin increased antifungal activity without cytotoxic effects on corneal tissues	[215]
Nanostructured lipid carriers (NLC)	Voriconazole	*C. albicans*	Anti-candidal hyphae formation	NLC sustained drug release, inhibited hyphae formation, and improved antifungal activities in vitro	[216]
Nanostructured lipid carriers (NLC)	Fluconazole	Fluconazole-resistant strains of *C. albicans, C. glabrata, C. parapsilosis*	Anti-fungal growth	NLC sustained drug release and improved fungicidal activities	[217]
Nanostructured lipid carriers (NLC) incorporated with poloxamer P407	Clotrimazole	*Candida albicans*	Anti-fungal growth	NLC-P407 provided an optimized drug delivery system with suitable viscosity for mucosal application and four fold more antifungal activities than Fungizone^TM^ (commercial antifungal drug) against *Candida albicans*	[218]
Nanostructured lipid carriers (NLC)	Miconazole	*Candida albicans*	Oral candidiasis	NLC enhanced drug release, improved local delivery of miconazole to the oral mucosa and improved antifungal effects than miconazole alone	[219]
Nanostructured lipid carriers (NLC)	Itraconazole	*S. brasiliensis, Candida albicans*	Skin fungal infection	NLC decreased transepidermal water loss, but improved drug delivery and antifungal activity	[220]
Nanostructured lipid carriers (NLC)	Ketoconazole	*C. neoformans*	Cryptococcal meningoencephalitis	NLC sustained drug release and improved antifungal effects in mouse brain tissues	[221]
Nanostructured lipid carriers (NLC) with Mediterranean essential oils	Clotrimazole	*Candida albicans, C. krusei, C. parapsilosis*	Skin candidiasis	NLC contained Mediterranean essential oils and enhanced membrane permeabilization and antifungal effects for treating topical candidiasis	[222]
Lipid nanocapsules (LNC)	Fluconazole	Fluconazole-resistant strains of *C. albicans, C. glabrata, C. krusei, C. tropicalis*	Anti-fungal growth	NLC provided the high drug carrying and controlled drug release system, enhanced antifungal activity to fluconazole-resistant *Candida* spp.	[223]
Lipid nanocapsules (LNC)	Amiodarone (AMD) and/or fluconazole (FLU)	*C. neoformans*	Anti-fungal growth	LNC displayed better antifungal effects, whether it was encapsulated with AMD, FLU, or AMD+FLU, than single usage of these two drugs in cryptococcal infected mice	[224]
